# Bipolar type I diagnosis after a manic episode secondary to SARS-CoV-2 infection: A case report

**DOI:** 10.1097/MD.0000000000029633

**Published:** 2022-08-05

**Authors:** Ambra D’Imperio, Jonathan Lo, Luca Bettini, Paco Prada, Guido Bondolfi

**Affiliations:** a Service of Forensic Psychiatry CURML, Geneva University Hospitals, Geneva, Switzerland; b Ross University School of Medicine, Miramar, FL, USA; c Service of Internal Medicine, Department of Medicine, Geneva University Hospitals, Geneva, Switzerland; d Service of consultative psychiatry and crisis intervention, Geneva University Hospitals, Rue Gabrielle Perret-Gentil 4, 1205 Geneva, Switzerland.

**Keywords:** bipolar type I disorder, manic episode, psychotic vulnerability, SARS-CoV-2, steroid treatment

## Abstract

Our objective is to provide awareness about psychotic vulnerability in patients infected with SARS-CoV-2 and to better understand the role of steroid withdrawal in manic episodes, especially with its common usage in respiratory disease caused by SARS-CoV-2.

**Patient concerns:**

We present the case of a patient who was hospitalized twice after discontinuing steroid therapy for SARS-CoV-2 infection and presented with a manic episode despite not having a psychiatric history.

**Diagnosis:**

The patient tested positive on a polymerase chain reaction test for SARS-CoV-2 and developed pneumonia. Other organic differential diagnoses such as encephalitis were also investigated and excluded. Manic episodes were diagnosed according to DSM-V criteria. Subsequently, the patient was diagnosed with type I bipolar disorder.

**Interventions:**

According to the protocols, supplemental oxygen therapy, prophylactic enoxaparin and intravenous (IV) steroids were administered. Steroid dosage was gradually reduced under supervision. During the acute mania, antipsychotics and benzodiazepines were administered.

**Outcomes:**

After discharge, the patient was admitted to the psychiatric consultation service. He first received mood stabilizer therapy and then received supportive psychotherapy.

**Lessons:**

Psychotic symptoms commonly occur after the discontinuation of high-dose steroid therapy; however, controlled tapering may prevent these side effects. Only a few cases have reported concomitant SARS-CoV-2 infection and manic episodes, often with an apparent relationship with steroid withdrawal syndrome. In this case, we considered psychotic vulnerability a condition that is often underestimated. In consideration of the SARS-CoV-2 pandemic, the case may represent an underlying trigger for psychotic decompensation, which, in concert with neuroinflammation, may induce a manic episode.

## 1. Introduction

Steroids have been recognized as a milestone in the pharmacological approach for patients with severe to moderate SARS-CoV-2 infection.^[1]^ As shown in different meta-analyses,^[2–4]^ high doses of intravenous steroids reduce mortality in patients with acute respiratory failure. Nevertheless, together with the positive effect in controlling and relieving inflammation, steroids are known to trigger psychiatric symptoms, such as psychosis and hypomania, in those with underlying potential after cessation..^[5]^ According to WHO guidelines regarding the use of corticosteroids in patients affected by severe COVID-19, the risk of developing neuropsychiatric symptoms is 0,81 but the level of evidence is still low.^[6,7]^ Because of the high neurotropism of the SARS-Cov-2 virus itself^[8,9]^ and the interplay between different cytokine cascades leading to generalized neuroinflammation, defining the origin of neuropsychiatric symptoms, such as mania, may represent a challenge, especially when administering a therapy that includes steroids. In our case, we present a patient finally diagnosed with type I bipolar disorder. Thus, the definition of a clear neuropsychiatric pattern in postacute COVID-19 remains undefined.^[10]^

## 2. Case presentation

We describe the case of a 57-year-old Caucasian man with no previous psychiatric medical history who experienced a manic episode postSARS-Cov-2 infection.

In January 2021, the patient visited the emergency department with complaints of shortness of breath, myalgia, and fatigue. On admission, he was hemodynamically stable with a regular heart rate of 74 bpm and blood pressure of 130/80 mm Hg. The patient presented with moderate anxiety and emotional distress. The body temperature was 36.6 °C and SpO2 was 89% on room air, with an RR of 16/m. A nasopharyngeal swab tested positive for SARS-CoV-2. Chest computed tomography revealed involvement of 35% of the total parenchyma, and a progressing bilateral multifocal interstitial pneumonia was diagnosed. Once admitted to our primary care COVID-19 units, supplemental oxygen therapy was necessary: with flow rates of 4 L/min oxygen flow on nasal cannula, a concentration of SpO_2_ of 98% was achieved. Prophylactic enoxaparin (40 mg/day) and prednisone (8 mg 3/day) were administered intravenously for 1 week. Steroid therapy was gradually diminished, targeting an individualized tapering of 3 days after prednisone therapy was suspended. The patient presented with bizarre behavior, extreme talkativeness, and reduced need for sleep. Secondary psychosis was suspected, but in the absence of withdrawal symptoms such as weakness, decreased appetite, nausea, vomiting, diarrhea, and abdominal pain, this iatrogenic hypothesis was excluded. The patient finally developed clear psychotic symptoms, such as grandiose delusions and tachypsychia that persisted for 3 days, necessitating an involuntary psychiatric hold. In the psychiatric emergency room, the patient tested negative on drug screening. Nevertheless, the patient also presented with a fluctuating cognitive pattern, anterograde amnesia, clouding of consciousness, and disorganized thinking. An electroencephalogram (EEG) reported abnormal findings such as a slowing lower right temporal rhythm theta (4–5 Hz) persistent with eye opening and sharp waves to the theta range in the right lower temporal lobe. Subsequently, a magnetic resonance imaging (MRI) of the brain was performed, aiming to exclude anatomical or SARS-CoV-2 etiologies. The report found a parietal enhancement in the V4 segment of the vertebral artery, suggesting endothelial inflammation. A lumbar puncture revealed a negative cerebrospinal fluid (CSF) laboratory panel for both infectious and autoimmune etiologies. During his stay, the patient underwent a battery of neuropsychological tests and completed several laboratory and clinical assessments to exclude COVID-19-related encephalopathy. A neurology consultation revealed no focal or meningeal symptoms. Both EEG and MRI revealed isolated abnormalities, but these findings were inconsistent with the diagnosis of subclinical SARS-CoV-2-related encephalitis. The only persistent clinical symptoms were an expansive mood, thinking acceleration, disjointed thoughts, and irritability. The Young Mania Rating Scale (YMRS) was used, with a score of 31, corresponding to a moderate rating. According to the Diagnostic and Statistical Manual of Mental Disorders, Edition 5 (DSM-V) criteria, the patient’s presentation was consistent with a manic episode (Table 1). As our patient mainly displayed manic symptoms, he received both pharmacological and standard psychiatric care during the trial, including access to calming environments and reduced stimulation, focused on reducing hypomanic symptoms. He was initially treated with 5 mg^2^/day of olanzapine and 2.5 mg lorazepam 3/day. The total duration of hospitalization was 15 days. After discharge, the patient returned to normal work and social activities. Compliance with medical treatment was described as satisfactory, and the patient was motivated to begin supportive psychotherapy.

**Table 1:** Timeline and clinical manifestations

Table 1	EEG	MRI	CSF	Neurological exam	Psychiatric status	Neuropsychological evaluation
Day 1 (steroid therapy introduction: *predni*sone iv 8 mg 3/day)				N/A	Anxiety	CAM (*Confusion Assessment Method*): 5/6
Day 5 (steroid therapy cessation)	Lower right temporal rhythms theta (4–5 Hz) persistent at opening eyes and sharp waves to theta range in right lower temporal lobe.	parietal enhancement, V4 segment of vertebral artery.	N/A	N/A	Mania: • Logorrhoea • Tachypsychia • Disjointed thoughts • Expansive mood • Delusions	
Day 15 (after antipsychotic treatment: 5 mg *olanzapine* 2/d and *lorazepam* 1 mg 3/d)	Normal pattern	No pathological findings	N/A	N/A	Resolution of manic symptoms	Brain fog: • Inability to concentrate • Memory loss • Clouding of consciousness

**Figure 1.**
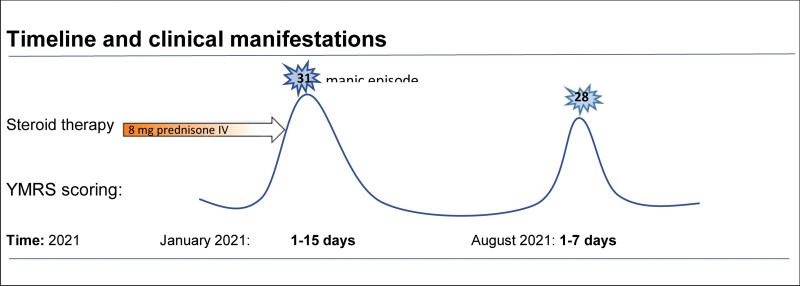
Evolution and findings on 15 days.

Nevertheless, 5 months after this episode, the patient developed a new manic manifestation. He was admitted to our hospital with grandiose delusions, disinhibited mood, logorrhea, flight of ideas, and severely disorganized thinking. After the administration of emergency antipsychotic treatment for a manic episode, the patient underwent a new mood-stabilizing treatment with quetiapine 400 mg 1/day. The duration of the second hospitalization was 7 days. (Fig. 1: Timeline and clinical manifestations) Since the patient was clinically stable and presented no significant abnormalities on serum analysis, no organic causes were suspected nor investigated. Control MRI of the brain was normal. The scoring of YMRS was 28, corresponding to a moderate manic episode rating. Other questionnaires, such as the Mood Disorder Questionnaire (MDQ), were submitted with a positive screening result for bipolar spectrum disorder. Moreover, a detailed family history revealed that the patient had a predisposition for mood disorders, with the mother and the brother each having an established diagnosis of unipolar depression. This clinical presentation with clear manic symptoms and a significant result in the YMRS, a normal MRI and lumbar puncture, and the absence of any drug withdrawal, confirmed the diagnosis of bipolar type I disorder. All psychopathological features were fulfilled, and the diagnosis was communicated to the patient. After several sessions of psychoeducation and clinical observations, the patient was discharged to continue supportive psychotherapy with a consultation service. On follow-up, the mood-stabilizing treatment of quetiapine was gradually augmented up to 500 mg 1/day, according to the Canadian Network for Mood and Anxiety Treatments (CANMAT) guidelines.^[11]^

### 2.1. Ethics approval and consent to participate

Written informed consent was obtained from the patient. This clinical case report describes the ethical rules applied at the Geneva University Hospital.

## 3. Discussion

This paper presents a case of bipolar disorder type I diagnosed after acute infection with SARS-CoV-2, and a differential diagnosis challenge. To our knowledge, other similar cases of manic manifestations during SARS-CoV-2 infection have been reported,^[12–14]^ but none have been associated with a diagnosis of type I bipolar disorder. Neuropsychiatric manifestations are generally mentioned as a common subgroup of symptoms, with no specific focus on manic episodes. Our report describes the steps made to diagnose bipolar disorder of type I when concurrent infection with SARS-CoV-2 occurs.

Thus, we emphasize the importance of paying attention to specific psychiatric manifestations, including manic symptoms. The patient showed good treatment compliance during his hospital stay. After 15 days, most symptoms disappeared. Physical and mental remission was achieved. Considering the different investigations performed, we detected significant alterations apart from some subjective feelings reported as “brain fog.” These residual symptoms, such as an overwhelming feeling of fatigue, unusual forgetfulness, and inability to focus on daily tasks, still had functional impact, but his mental status was considered stable. After discharge, the patient was referred to a psychiatric outpatient clinic for follow-up and recovery. Our findings suggest that while some long-term cognitive, emotional, and neuropsychological complications may represent a possible worsening effect on SARS-CoV-2 prognosis, the manifestation of psychiatric consequences in an early postrecovery stage is yet to be elucidated. Many studies have focused on long-term sequelae^[15]^ despite the presence of psychiatric symptoms in the acute stage. Defining the colloquial term “Covid-fog” clinically as a form of brain fog, and thus a mild type of cognitive impairment (MCI), may help it be understood as a temporary and reversible neuropsychiatric manifestation, as described in our clinical case. In addition, both a psychiatric predisposition and the influence of a neuroinflammatory trigger may be considered 2 identifiable criteria for defining postacute Covid syndrome.^[16,17]^ Possible explanations include an alteration in regulatory immune cells in the brain, which has been shown in patients diagnosed with bipolar disorder type II, leading to psychotic vulnerability.^[18]^ Moreover, a high sensitivity to a neuroinflammatory trigger such as SARS-CoV-2, may reveal a preexisting pro-inflammatory pattern, which is unknown when the patient does not have a psychiatric history.^[19]^ In addition, some studies suggest that the neurophysiological activity is altered in some brain regions such as the prefrontal, central, occipital, and temporal lobes in patients suffering from a bipolar mood disorder, with a major disorganization in the right anterior hemisphere and a positive correlation between the qEEG (quantitative Electroencephalogram) reading and the presence of family history of bipolar disorder.^[20,21]^ Thus, qEEG and SPECT (Single-photon emission computer tomography) brain imaging may provide more data in order to support or not the diagnosis and to elucidate the neurofunctional basis of the cortical areas that might be affected. A potential causal relevance of steroid withdrawal is plausible, but more research is needed to better understand the early phase manifestations of mood disorders and to prevent pure psychiatric ebbing. In the future, more case studies are encouraged to improve the current treatment and to explore the long-term psychopharmacological needs, such as for the prevention of relapses.^[22]^

## 4. Conclusions

We conclude that psychotic vulnerability secondary to a neuroinflammatory insult is a risk factor in people affected with SARS-CoV-2 infection and that careful attention is needed, especially when administering steroids. Our case suggests that psychiatric manifestations should be monitored not only in postacute Covid syndrome but also in the early stages of SARS-CoV-2 infection. Developing a manic episode after SARS-CoV-2 infection may be a clue for detecting preexisting psychotic vulnerability, leading to a bipolar type I diagnosis.

## Author contributions

ADI: conceptualization, data collection, writing-original draft preparation

LB: drafting and critical revision of the article

JL: drafting and critical revision of the article, reviewing, and editing

PP: critical revision of the article, supervision

GB: critical revision of the article, supervision
